# Molecular docking analysis of aldose reductase with herbal compounds from selected siddha medicine for anti-cataract treatment

**DOI:** 10.6026/973206300210559

**Published:** 2025-03-31

**Authors:** Vinodini Ramamoorthy, Saraswathi Balasubramanian, Siva Annamalai, Deepa Ravichandran, Vinu Bharathi Balasubramaniam, Shunmugaram Shenbagaraj

**Affiliations:** 1 Department of Clinical Research, Siddha Regional Research Institute, Central Council Research in Siddha, Ministry of Ayush, Government of India, Kuyavarpalayam, Puducherry, India; 2 Department of Clinical, Siddha physician, Om Muruga Siddha clinic, Thanjavur, India

**Keywords:** Cataract, kan noigal, siddha medicine, eye diseases, epalrestat

## Abstract

Cataracts are a major cause of blindness and it is due to protein aggregation in the eye lens that is accelerated by aldose reductase (AR)-induced sorbitol accumulation. Therefore, it is of interest to evaluate the anti-cataract potential of six phytochemicals from Siddha medicinal herbs (carinol, voacangine, berberine, piperine, sesamin and isoamericanin A) using molecular docking analysis with human aldose reductase (PDB ID: 4GCA) where epalrestat is used as the standard compound. Analysis shows that berberine, piperine, sesamin and carinol have optimal binding interactions with aldose reductase, performing similar to the standard drug epalrestat having estimated free energy values of -10.37 kcal/mol and -10.13 kcal/mol, respectively for further consideration. Thus, the traditional use of these herbs in Siddha medicine as a potential, affordable and non-surgical cataract-preventative agent is shown.

## Background:

Cataracts, characterized by the clouding of the eye's lens, are a leading cause of vision loss and blindness, primarily caused by aging but also by factors such as congenital conditions, eye injuries, inflammation and diseases like glaucoma and diabetes. Cataract development is also associated with steroid use and certain radiation exposures. As global life expectancy increases, the prevalence of cataracts may raise, which leads to 2.2 billion individuals with vision impairment, among that one billion cases are either untreated or preventable globally. World Health Organisation (WHO) estimates that 36% of distance vision impairment and 17% of cataract-related vision impairment only receive an appropriate intervention in global population. Despite that, the cataracts can be curable by surgery but also unattainable in many nations [1, 2]. In India, Cataract remains the major cause of blindness (66.25%) and visual impairment (71.25%) in individual's age around 50 and older, even though with the high cataract surgical coverage rate (93.2%). Due to the unique benefits of modern intraocular lenses with advanced materials and optical properties, surgery has become the primary treatment for cataracts. However, surgery has the complications, like incision infections, posterior capsular rupture, capsular contraction syndrome, intraocular lens dislocation and dry eyes [5]. Research studies revealed that uncontrolled systemic health conditions, financial difficulties, lack of perceived need for surgery and fear in surgical procedures are the barriers that inhibit the elderly and rural populations for undergo cataract surgery [4]. The out of pocket expenses for pediatric cataract surgery in one eye ranged from Rs. 4,722 ($122) to Rs. 18,537 ($475), that reduces the treatment accessibility among public in developing nations [3, 5]. Since the cataract is unpreventable, but its development may delayed by reducing cigarette smoking, alcohol consumption and exposure of ultraviolet light. Therefore, it needs to explore medications for the prevention and treatment of cataract is of great importance. In Siddha system of medicine *Akattiyar Nayana Viti* and *Nakamuni Nayana Viti* precisely mentioned eye diseases under 96 types of *Kan noikal* along with therapeutic management and lifestyle modifications. Jeyavenkatesh *et al.* provided an in-depth analysis and clinical correlation of *Kan noikal* (eye diseases) with modern diagnostic perspectives. They distinctly identified and described conditions such as *Neeraezhu kaasam* (Retinal cataract), *Neela kaasam* (Blue opaque cataract), *Kumari kaasam* (Chronic cataract), *Paravai* Poo (Opaque crystal cataract), *Silettuma kaasam* (White cataract) and *Pittha kaasam* (Yellow cataract). The treatment modalities are categorized into *Deva Maruthuvam*, *Maanidar Maruthuvam* and *Asura Maruthuvam* based on the disease's signs and symptoms, severity and prognosis.

In *Deva Maruthuvam* and *Maanidar Maruthuvam*, therapies such as herbal extracts, ghee-based collyriums, oil baths, poultices and fomentations - often incorporating mineralo-metallic ingredients - are extensively employed. In contrast, *Asura Maruthuvam* involves surgical interventions, including excision, pricking, peeling, venesection, leech therapy and cauterization. These procedures utilize specialized instruments such as *Vilisam*, *Pulladi*, *Kakkai Kaal*, *Piruma*, *Anjanak Koel* and *Kariak Koel*. Additionally, the pre- and post-operative protocols, along with medications specific to surgical procedures, are meticulously detailed in *Akattiyar Nayana Viti*. Cataract, being one such condition, has been comprehensively addressed within it [6, 7]. Cataracts involve lens clouding caused by protein and fiber changes, with osmotic expansion that accelerates the disease progression. Aldose reductase, an enzyme implicated in cataract development, converts glucose to sorbitol, leading to its accumulation in lens cells and causing osmotic stress and damage; inhibiting this enzyme may prevent or delay cataract formation. Epalrestat, a renowned aldose reductase inhibitor, was selected as standard to evaluate the efficacy of the herbal samples [8]. Binding of phytocomponents with the core amino acids (Trp20, Val47, Tyr48, Trp79, His110, Trp111, Thr113, Phe122, Gln183, Tyr209, Ala299 and Leu300.) of the target by forming hydrogen bond will inhibit the activity of the enzyme aldose reductase, thereby will delay sorbitol accumulation and contributes to delayed cataract development. *Carissa spinarum*, *Tabernaemontana divaricata*, *Coptis teeta*, *Piper longum* and *Trianthema decandra* are among the Siddha medicinal herbs exclusively mentioned for their use in treating eye diseases, with potential anti-cataract properties [9]. Therefore, it is of interest to explore their anti-cataract efficacy by evaluating their ability to inhibit aldose reductase activity so as to delay the progression of cataracts.

## Materials and Methods:

## Study drug:

The study evaluated six phytochemicals derived from medicinal herbs traditionally used in Siddha medicine for eye diseases, such as Carinol from *Carissa spinarum*, Voacangine from *Tabernaemontana divaricata*, Berberine from *Coptis teeta*, Piperine and Sesamin from Piper longum and Isoamericanin A from *Trianthema decandra* that are detailed in Table 1. Additionally, Epalrestat was used as a standard drug.

## Target protein preparation:

The target protein, Human Aldose Reductase (PDB ID: 4GCA), was retrieved from the Protein Data Bank and prepared for docking by removing water molecules, ligands and non-essential heteroatoms, followed by energy minimization to optimize the structure [15]. Key active site residues associated with cataract formation - Trp20, Val47, Tyr48, Trp79 and His110 were identified as primary binding sites for ligand interactions. The 3D crystal structure of the target protein is shown in Figure 1 (see PDF).

## Ligand preparation:

Each selected phytochemical was prepared for docking by obtaining its 2D and 3D structures from databases or molecular modelling software, followed by energy minimization to ensure stable conformations and reduced steric hindrance. Each ligand was then parameterized with appropriate partial charges and rotatable bonds to enable flexible interactions with the target protein. The structured of ligands are shown in Figure 2 (see PDF) and their properties were tabulated in Table 2.

## Docking procedure:

Docking simulations were conducted using AutoDock tools to evaluate the binding interactions between the target protein and each ligand. A grid box was centered on key active site residues to confine docking to relevant regions. Parameters, including binding affinity (ΔG), inhibition constant (Ki) and interaction surface, were calculated for each ligand [16, 17]. The binding interactions and energy values were then analyzed and compared with those of Epalrestat to determine the relative binding efficacy of each compound.

## Results:

The molecular interactions showed that compounds like berberine, piperine, sesamin and carinol exhibited 8 to 10 viable interactions with the residual amino acid present in the target aldose reductase enzyme whereas Epalrestat showed 8 interactions. Carinol exhibited the strongest binding affinity with an estimated energy of -10.13 kcal/mol and an inhibition constant (Ki) of 37.38 nM, showing 10 key interactions with Trp20 and His110. Sesamin followed closely with a binding energy of -10.37 kcal/mol and a Ki of 25.25 nM, indicating its effectiveness with 8 interactions. Piperine and Berberine also showed good binding profiles, both with 8 interactions, while Voacangine and Isoamericanin A had less interaction. Notable amino acids involved included Trp20, Val47 and Tyr48, which play essential roles in the enzyme's activity. The summary of the molecular docking studies of Phytochemicals against Human Aldose reductase enzyme (PDB) - 4GCA are tabulated in Table 3 and Amino acid Residue Interaction of Lead against Human Aldose reductase enzyme (PDB) - 4GCA are outlined in Table 4. Likewise the Docking poses of phytochemicals with Human Aldose reductase enzyme, 2D interaction plot analysis of Phytochemicals and the Hydrogen bond plotting with core amino acid Analysis of Phytochemicals are depicted in Figure 3 (see PDF), Figure 4 (see PDF) and Figure 5 (see PDF).

## Discussion:

Molecular docking is a computational tool used to predict the binding potential of small molecules, such as phytochemicals from medicinal plants, to specific targets like enzymes. In this study, bioactive compounds including Carinol, Voacangine, Berberine, Piperine, Sesamin and Isoamericanin A were selected from *Carissa spinarum*, *Tabernaemontana divaricata*, *Coptis teeta*, *Piper longum* and *Trianthema decandra*, respectively, to evaluate their *In-silico* aldose reductase inhibition activity. The results revealed that Berberine, Piperine, Sesamin and Carinol demonstrated strong binding interactions with the core amino acids of aldose reductase, performing either on par with or better than the standard drug Epalrestat. Among these, Sesamin exhibited the highest binding affinity, with a calculated free energy of binding of-10.37 kcal/mol, surpassing Epalrestat's-9.90 kcal/mol, suggesting greater potential effectiveness in preventing cataract formation. This strong binding is further reinforced by Sesamin's anti-inflammatory and antioxidant properties that help to reduce oxidative stress a major factor in cataractogenesis and promote overall lens health [21].

The multiple role of Sesamin as an aldose reductase inhibitor and antioxidant may offer a protective effect against cataracts, particularly in populations vulnerable to oxidative stress, such as the elderly and diabetics. Furthermore, Berberine, Piperine and Carinol are phytochemicals with promising anti-cataract potential, primarily attributed to their antioxidant [10, 18, 19], anti-inflammatory [10, 18, 20] and aldose reductase inhibitory properties [18, 19, 22]. These compounds help protect the lens from oxidative stress, a key contributor to cataract formation. Additionally, by inhibiting the enzyme aldose reductase, they prevent the accumulation of sorbitol in the lens, a mechanism particularly crucial in managing diabetic cataracts. Research studies have revealed that the methanolic extract of *Piper longum* Linn effectively neutralizes free radicals, thereby preventing lens opacification [2323]. *Trianthema decandra* has traditionally been used to treat conditions such as corneal ulcers, night blindness and bacterial infections. Its methanolic extract (METD) has shown anti-cataract potential by maintaining GSH levels and mitigating oxidative stress [24]. *Coptis teeta*, which contains berberine, exhibits antioxidant, -inflammatory and anti-trachoma properties [25]. Meanwhile, *Tabernaemontana divaricata* protects against selenite-induced cataractogenesis by preserving lenticular GSH, maintaining calcium homeostasis and safeguarding lens proteins from proteolysis [26]. Collectively, these plants demonstrate significant therapeutic potential for cataract prevention through their antioxidant and anti-inflammatory properties.

Multitudinous scientific studies explore the efficacy of medical plants in the context of cataract prevention by inhibiting aldose reductase and free radical scavenging. *Gacche and Dhole* and *Upadhyaya* *et al.* highlighted the anti-cataract activity of *Terminalia arjuna* *Upadhyaya*, *Aegle marmelos*, *Withania somnifera*, *Boswellia serrata*, *Moringa oleifera*, *Tinospora cordifolia*, *Azadirachta indica*, *Emblica officinalis*, *Terminalia bellirica*, *Terminalia chebula*, *Curcuma longa* and *Camellia sinensis* by their aldose reductase inhibition and oxidative stress inhibitory activity [27, 28]. Ji *et al.* exhibited the *in-vivo* aldose reductase inhibition activity of *diosgenin* an active moiety of *Dioscorea villosa* in a galactosemic rat model, underscoring its potential as an anti-cataract agent [8]. Collectively, these studies emphasize the significate role of medicinal herbs in cataract management. According to Siddha principles, cataract is a pathological condition resulting from vitiated *Iyam* and primarily occurs during *Iya kaalam* (the geriatric age group). The herbs recommended for treating cataracts, which are predominantly bitter in taste, have the ability to reduce vitiated *Iyam* and prevent cataract progression due to their anti-cataract, antioxidant and anti-inflammatory properties. These properties, reinforced by molecular docking studies, indicate that compounds in this study have the potential to delay cataract progression and may serve as promising non-surgical treatments for cataracts. As a result, the utilization of these phytocompounds could provide a non-invasive and cost-effective alternative or complement to cataract surgery, particularly in resource-constrained settings where access to surgical interventions is limited.

## Implications for future research:

This study emphasizes the need for extensive *in-vitro* and *in-vivo* research, along with clinical trials, to validate the efficacy and safety of these phytochemicals. While *Tabernaemontana divaricata* and *Trianthema decandra* are traditionally used to treat various eye conditions, the active components Voacangine and Isoamericanin A identified in this study showed limited efficacy compared to the standard. Further research is essential to explore and evaluate other bioactive compounds from these plants for their potential anti-cataract properties. Additionally, future studies should investigate synergistic interactions to enhance therapeutic effects, assess bioavailability and toxicity and determine their feasibility as alternative or complementary treatments for cataracts.

## Conclusion:

The molecular docking analysis of phytochemicals derived from Siddha medicinal herbs showed optimal binding interactions with aldose reductase, suggesting promising anti-cataract activity. The high binding affinities of compounds such as sesamin and carinol suggest their potential as non-surgical, preventive solutions for cataract development, particularly in high-risk populations. This study not only validates the traditional use of these herbs in Siddha medicine but also highlights their phytochemicals as promising prospects for further pharmacological research in cataract prevention.

## Ethical approval:

The authors declare that due to an *In-silico* analysis this study does not need any ethical approval.

## Consent to participate:

Not applicable

## Consent to publish:

Not applicable

## Author contributions:

All authors have equally contributed in the manuscript preparation like concept, designing and collection of the data, as well as the writing and revision of the article.

## Funding:

The authors declare that no funds, grants, or other support were received during the preparation of this manuscript.

## Availability of data and materials:

All the data's are available with authors upon reasonable request

## Source of funding:

No funds, grants, or other support was received for this work.

## Figures and Tables

**Table 1:** Medicinal herbs and their selected phytocomponents for docking

**S. No**	**Medicinal herbs**		**Indication as per Siddha literature**	**Phytochemicals**
	**Botanical Name**	**Vernacular Name**		
1.	Carissa spinarum	Cirukalapu	Cataract, Corneal opacity, Crushed water drops applied before sunrise, to relieve symptoms of eye diseases.	Carinol [10]
2.	Tabernaemontana divaricate	Nantiyavattampu	Cataract, Diseases of pupil.	Voacangine [11]
3.	Coptis teeta	Pitarokini	Cataract, Diseases of eyelid, Decoctions for eye wash helps to cleanse and prevent Eye diseases, Root powder - Oil bath used to prevent eye diseases.	Berberine [12]
4.	Piper longum	Tippili	Helps to prevent eye disease.	Piperine [13], Sesamin [13]
5.	Trianthema decandra	Caranaiver	96 types of Eye diseases.	Isoamericanin A [14]

**Table 2:** Ligand properties of the compounds selected for docking analysis

**Compound**	**Molar weight g/mol**	**Molecular Formula**	**H Bond Donor**	**H Bond Acceptor**	**Rotatable bonds**
Carinol	618.8 g/mol	C_39_H_42_N_2_O_5_	5	5	12
Voacangine	368.5 g/mol	C_22_H_28_N_2_O_3_	1	4	4
Berberine	336.4 g/mol	C_20_H_18_NO_4_	3	0	4
Piperine	285.34 g/mol	C_17_H_19_NO_3_	0	3	3
Sesamin	354.4 g/mol	C_20_H_18_O_6_	0	6	2
Isoamericanin A	328.3 g/mol	C_18_H_16_O_6_	3	6	4
Epalrestat	319.4 g/mol	C_15_H_13_NO_3_S_2_	1	5	4

**Table 3:** Summary of the molecular docking studies of phytochemicals against human aldose reductase enzyme (PDB) - 4GCA

**Compound**	**Est. Free Energy of Binding**	**Est. Inhibition Constant, Ki**	**Electrostatic Energy**	**Total Intermolecular Energy**	**Interact. Surface**
Carinol	-10.13 kcal/mol	37.38 nM	-0.03 kcal/mol	-11.44 kcal/mol	852.146
Voacangine	-8.31 kcal/mol	808.26 nM	-0.07 kcal/mol	-9.00 kcal/mol	787.447
Berberine	-8.87 kcal/mol	317.26 nM	-0.11 kcal/mol	-9.36 kcal/mol	737.756
Piperine	-9.96 kcal/mol	49.94 nM	-0.01 kcal/mol	-10.47 kcal/mol	715.148
Sesamin	-10.37 kcal/mol	25.25 nM	-0.04 kcal/mol	-10.91 kcal/mol	839.454
Isoamericanin A	-9.02 kcal/mol	244.57 nM	-0.11 kcal/mol	-9.30 kcal/mol	759.975
Epalrestat	-9.90 kcal/mol	55.17 nM	-0.85 kcal/mol	-11.24 kcal/mol	725.143

**Table 4:** Amino acid residue interaction of lead against human aldose reductase enzyme (PDB) - 4GCA

**Compounds**	**Interaction**	**Amino Acid Residues**
Carinol	10	20	48	77	110	111	113	115	122	159	160	183	209	219	298	299	300	303	309	311
		TRP	TYR	LYS	HIS	TRP	THR	PHE	PHE	SER	ASN	GLN	TYR	TRP	CYS	ALA	LEU	CYS	TYR	PHE
Voacangine	6	20	47	48	49	111	121	122	219	300
		TRP	VAL	TYR	GLN	TRP	PHE	PHE	TRP	LEU
Berberine	8	20	47	79	111	113	115	122	219	299	300	303	309	310	311
		TRP	VAL	TRP	TRP	THR	PHE	PHE	TRP	ALA	LEU	CYS	TYR	PRO	PHE
Piperine	8	20	48	79	110	111	113	115	122	219	298	300	303	309
		TRP	TYR	TRP	HIS	TRP	THR	PHE	PHE	TRP	CYS	LEU	CYS	TYR
Sesamin	8	20	43	48	110	111	122	160	183	209	210	219	260	262	298	300
		TRP	ASP	TYR	HIS	TRP	PHE	ASN	GLN	TYR	SER	TRP	ILE	LYS	CYS	LEU
Isoamericanin A	5	20	47	48	49	111	121	122	219	300	303	309
		TRP	VAL	TYR	GLN	TRP	PHE	PHE	TRP	LEU	CYS	TYR
Epalrestat (Standard drug)	8	20 TRP	47 VAL	48 TYR	79 TRP	111 TRP	113 THR	115 PHE	122 PHE	219 TRP	300 LEU	303 CYS
